# Social-ecological analysis of timely rice planting in Eastern India

**DOI:** 10.1007/s13593-021-00668-1

**Published:** 2021-02-18

**Authors:** Anton Urfels, Andrew J. McDonald, Gerardo van Halsema, Paul C. Struik, Pankaj Kumar, Ram K. Malik, S. P. Poonia, Balwinder-Singh, Deepak K. Singh, Madhulika Singh, Timothy J. Krupnik

**Affiliations:** 1 Sustainable Intensification Program, International Maize and Wheat Improvement Centre (CIMMYT), South Asia Regional Office, Khumaltar, Lalitpur, Nepal; 2 Water Resources Management Group, Wageningen University & Research, Wageningen, Netherlands; 3 Centre for Crop Systems Analysis, Wageningen University & Research, Wageningen, Netherlands; 4 Section of Soil and Crop Sciences, School of Integrative Plant Sciences, Cornell University, Ithaca, NY USA; 5 International Maize and Wheat Improvement Centre, NASC Complex, New Delhi, India; 6 Sustainable Intensification Program, International Maize and Wheat Improvement Centre (CIMMYT), Dhaka, Bangladesh

**Keywords:** Sustainable agriculture, Climate-resilient agroecosystems, Eastern Gangetic Plains, Mixed methods, Rice-wheat system, Machine learning, Groundwater, Monsoon onset, Sowing date, Landscape level

## Abstract

Timely crop planting is a foundation for climate-resilient rice-wheat systems of the Eastern Gangetic Plains—a global food insecurity and poverty hotspot. We hypothesize that the capacity of individual farmers to plant on time varies considerably, shaped by multifaceted enabling factors and constraints that are poorly understood. To address this knowledge gap, two complementary datasets were used to characterize drivers and decision processes that govern the timing of rice planting in this region. The first dataset was a large agricultural management survey (rice-wheat: *n* = 15,245; of which rice: *n* = 7597) from a broad geographic region that was analyzed by machine learning methods. The second dataset was a discussion-based survey (*n* = 112) from a more limited geography that we analyzed with graph theory tools to elicit nuanced information on planting decisions. By combining insights from these methods, we show for the first time that differences in rice planting times are primarily shaped by ecosystem and climate factors while social factors play a prominent secondary role. Monsoon onset, surface and groundwater availability, and land type determine village-scale mean planting times whereas, for resource-constrained farmers who tend to plant later *ceteris paribus*, planting is further influenced by access to farm machinery, seed, fertilizer, and labor. Also, a critical threshold for economically efficient pumping appears at a groundwater depth of around 4.5 m; below this depth, farmers do not irrigate and delay planting. Without collective action to spread risk through synchronous timely planting, ecosystem factors such as threats posed by pests and wild animals may further deter early planting by individual farmers. Accordingly, we propose a three-pronged strategy that combines targeted strengthening of agricultural input chains, agroadvisory development, and coordinated rice planting and wildlife conservation to support climate-resilient agricultural development in the Eastern Gangetic Plains.

## Introduction

### Timely crop planting: a critical decision point for agroecosystem resilience

Attaining food security in the densely populated Eastern Indo-Gangetic Plains—a global poverty hotspot—requires the negotiation of trade-offs between productivity, risk, and the ecological footprint of agriculture, a challenge further compounded by the impacts of climate change (Park et al. 2018; Struik and Kuyper 2017; Ortiz et al. 2008). Building agroecosystem resilience—i.e., the capacity to maintain core functions in the light of environmental and market shocks (Nystrom et al. 2019)—and thus maintaining high levels of crop productivity are often predicated on timely crop planting and harvesting (Singh et al. 2019). Timely planting aligns crop cycles with favorable climate conditions resulting in higher and generally more stable yields. Specifically, timely crop planting raises system productivity by (a) mitigating risks of yield losses caused by pushing crop growth into periods of sub-optimal or extreme weather conditions such as cold and heat waves, drought, or flooding; (b) increasing resource use efficiencies; and (c) allowing for more crops to be grown per year on the same land (Acharjee et al. 2019). While several studies have analyzed optimal time windows for planting, agroecosystem characteristics and farmers’ decision processes that enhance or limit the potential to plant crops during optimal time windows have received less attention (Acharjee et al. 2019; Singh et al. 2019; Mingxia et al. 2020).

With approximately 400 million people and a population density of more than 1000 people per km^2^, the Eastern Gangetic Plains encompass parts of Northeastern India, eastern Nepal, and Bangladesh. High incidence of poverty and food insecurity, as well as a primary dependence on agriculture, make it a priority location for achieving the Sustainable Development Goal 1: No Poverty, and Sustainable Development Goal 2: Zero Hunger (Jat et al. 2020). In this region, farmers predominantly grow rice during the summer monsoon, often followed by a wheat crop in the dry winter season. But, erratic monsoon patterns increasingly cause both floods and droughts in close spatio-temporal proximity, threatening farmers’ productivity. Research over the last decades has shown that timely planting of both rice and wheat is one of the most important response options that farmers in the region have to build resilient agroecosystems amidst changing climate regimes (Keil et al. 2019; Ortiz et al. 2008; Singh et al. 2019). At the system level of rice-wheat cropping patterns in South Asia, timely planting of rice facilitates the efficient use of monsoon season rainfall and, just as importantly, planting of wheat within the first 3 weeks of November. The latter assures higher yield potential by avoiding both season-long and terminal heat stress during grain filling (Singh et al. 2019). Due to the cascading influence of rice management on subsequent crops like wheat in the annual rotation, our study focuses on rice planting.

**Fig. 1:**
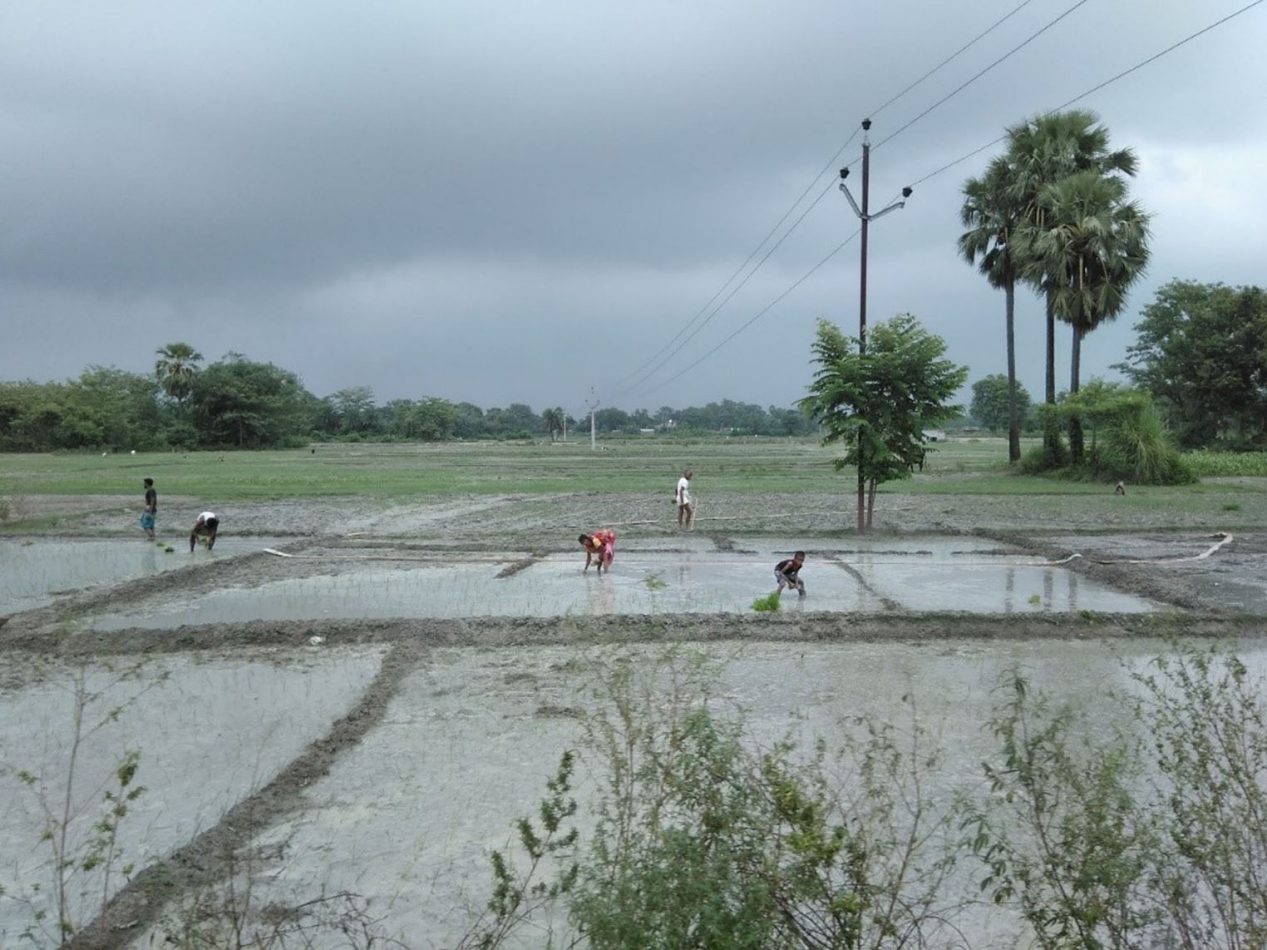
Farmers transplanting rice seedlings on July 31, 2017, in the Eastern Gangetic Plains (Bihar, India). Source: Anton Urfels

Rice planting is typically a two-step process as nurseries are first planted to raise seedlings that are then uprooted and transplanted into main fields (see Fig. 1). For simplicity, and because the timing of the two activities is highly correlated, we refer to the process of nursery establishment and subsequent transplanting as “planting.” Conversely, wheat tends to be broadcast sown after rice harvest following tillage to prepare fields. But, a delayed rice crop can push back the timely planting of wheat and other dry season crops. As the planting time of rice is a keystone to the productivity of this cropping sequence, the lack of knowledge of the factors that increase the ability of farmers to adopt timely planting hinders effective and targeted agricultural development programming in the region, particularly in light of the high levels of social and agroecological diversity. We therefore hypothesize that farmers’ capacity to plant on time is unequal and shaped by a host of binding constraints and enabling factors that are, at present, insufficiently understood.

### Farmers’ capacity to adjust planting dates: a systems’ perspective

In this study, we draw on social-ecological systems research as it pertains to resilience theory. We considered the work of Lescourret et al. (2015) and distinguished two different types of factors: (a) ecosystem factors that operate largely at the landscape level but exert influence on individual farmers’ ability to plant on time and (b) social system factors (henceforth “social factors”) that operate at the village and household scales and affect farmers’ decisions regarding planting times.

Ecosystem factors include dynamic factors that change from year to year such as the onset of the monsoon, and pest and disease pressures, but also static factors that remain relatively constant over time such as pre-monsoon ground and surface water availability, and land types (e.g., the position of a plot within the drainage system where water tends to accumulate in lowlands or to runoff in upland areas).

Social factors are mainly associated with input and resource availability. Timely planting requires readily available seed and fertilizer, tractors for land preparation, and irrigation (e.g., mostly with groundwater, but sometimes also in the form of canal water), in addition to labor and capital to pay for crucial operations. These factors are influenced by household resource endowment, availability of farm machinery, market access, and many others. Since ecosystem factors operate largely at the landscape level, they can be regarded as boundary conditions for individual villages and households. Social factors at village and household levels then shape responses therein.

This study identifies and characterizes the main factors and decision processes that influence capacity to achieve timely rice planting in the Eastern Gangetic Plains. We deployed a mixed-methods approach to understand factors associated with the timely planting of rice—a key indicator of agroecosystems’ resilience in the Eastern Gangetic Plains, particularly in the light of progressive climatic change. Specifically, we studied how social-ecological characteristics differ across early, medium, and late rice planters. We worked on the assumption that timely planting means early planting in most cases, as indicated by overall yield benefits to the rice-wheat system (Ortiz et al. 2008; Singh et al. 2019). We present an approach in which we combine insights from two unique datasets, a detailed discussion-based dataset and a big picture survey. We analyzed each dataset through novel methods and used the results from the detailed dataset to complement and inform interpretation of results from modern data-mining techniques that we used to analyze the big picture dataset. In Section 2, we sketch out the broad research design and theoretical considerations and delineate how we addressed these considerations. This section is designed to allow a better understanding of our contributions to developing an innovative mixed-methods approach for gaining insights for resilience in social-ecological system from cross-sectional case studies (Bodin et al. 2019). In Section 3, we present the results and (a) discuss the social-ecological factors that the study revealed as most important for timely planting, (b) assess the value of our mixed-methods approach to study complex social-ecological systems at regional scale, and (c) propose a practical strategy for building resilient rice-wheat systems in the Eastern Gangetic Plains through timely planting.

## Materials and methods

### Datasets: households selection, sampling strategy, and data collection

**Fig. 2:**
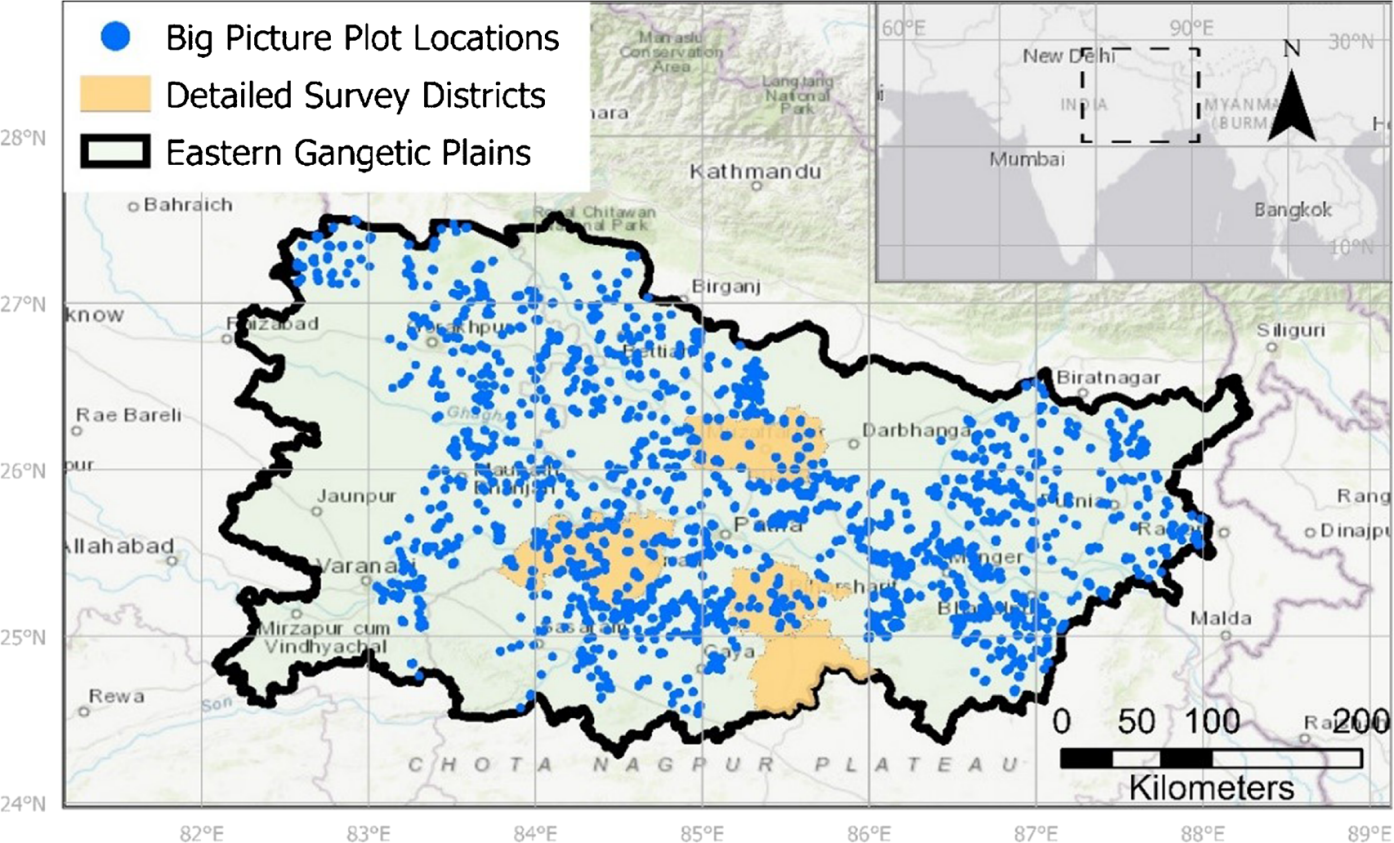
Map of the study area showing the locations of the plots of the big picture survey and the districts in which the detailed surveys were conducted

We used two complementary datasets in this study that we call “big picture” dataset and “detailed” dataset. The big picture dataset is a farmer survey developed for crop diagnostics at the regional scale (rice-wheat: *n* = 15,245, out of which rice: *n* = 7597) for the 2017–2018 rice-wheat season in the state of Bihar and neighboring parts of Uttar Pradesh. It was collected in 2017–2018 through a collaborative effort between the Cereal Systems Initiative for South Asia (CSISA; www.csisa.org) and the Indian Council of Agricultural Research and their network of Krishi Vigyan Kendra (KVK) offices that bring scientific expertise to the district level. We first selected 39 districts with 30 districts in Bihar State and nine in adjacent areas of Uttar Pradesh State. In each district, we randomly selected 30 villages. Next, a random draw from voter rolls was used to identify seven farm households to survey within each village. This produced a total of 210 household samples in each district (Fig. 2) and a total of 8190 target sampling households for each crop. Some data points had to be discarded during data cleaning, producing the total survey size of 15,245. Survey responses were elicited for the largest rice field managed by each household. The survey was designed to elucidate patterns of production practices and yield outcomes across the region. Following geo-tagging of farmers’ largest field, we elicited data describing crop management practices, bio-physical site characteristics, and causes of crop stress. Farmers were also queried regarding their level of market integration and orientation and social and household characteristics.

The detailed dataset is a survey based on focus group discussions with farmers in Bihar that characterizes farmers’ perceptions of factors affecting the timing of rice establishment in their villages, with associated scoring of how much each identified factor affects the timing of planting and productivity on their own farms (*n* = 112 farmers in 22 focus groups with, on average, 5 farmers per focus group). For the detailed dataset, we subsampled—out of the big picture sample frame—five randomly chosen households in five villages in three study areas that represent different key agroecological zones (Fig. 2). This relatively small subsample was chosen to provide a more qualitative in-depth perspective across social-ecological gradients in the region to help explain and inform results of the quantitative analysis of the big picture survey. The three study areas represent the two biggest agroecological zones of Bihar, as well as one drought-prone area with less reliable access to irrigation: (1) Muzaffarpur/Samastipur (good rainfall and aquifers, partial canal irrigation), (2) Bhojpur/Buxar (located at the tail end of a canal irrigation scheme with good aquifers and heavy soils), and (3) Nalanda/Jehanabad (a small canal irrigation scheme with poorer and more heterogeneous aquifers, lighter soils, and hence, more drought prone). The villages were chosen by local agricultural experts who were asked to select villages that represent the variation in social-ecological conditions within the study areas to ensure that inter-village variability is controlled for. The households within each village were chosen at random to control for intra-village diversity, and participation of different socio-economic groups was observed in the focus group discussions.

The goal of the detailed survey was to elucidate a system description of planting date decisions based on the logic and language of the farmers (i.e., an emic perspective). To achieve this, we facilitated the group of the selected survey respondents to construct a causal diagram in each village (Dorward et al. 2007). Often, several other interested individuals joined the discussions which we allowed to source information from the largest possible group. We then placed a flipchart on the ground and wrote down the two items: (a) nursery establishment and (b) transplanting in Hindi and English in the middle of the flipchart. We then told the participants that we sought to understand the factors that govern the timing of the two activities and asked them to complete the flipchart by brainstorming all possible factors that could drive the timing of rice planting. Arrows were then placed between factors indicating cause and effect. This process was facilitated by local staff in Hindi and other local languages as required. After this exercise, we asked the previously randomly selected 5 households in each focus group (these all participated in the big picture survey and were randomly chosen from voting lists) in each village to individually score each cause and effect relationship from 0 to 10 depending on the degree that, on average across households, it mattered for their own management of planting dates over the last 5 years (Dorward et al. 2007).

### Methodological approach: mixed-methods analysis of complex agroecological systems at the regional scale

The advantage of a mixed-methods approach is that they allow researchers to complement quantitative datasets with rich and contextual data (Bodin et al. 2019). We achieve this by combining the big picture and detailed dataset.

#### Big picture dataset: machine learning analytics for household survey data

The big picture dataset adds quantitative and spatial dimensions that could not be achieved with detailed surveys. But, quantitative modeling to identify factors influencing rice establishment with the larger dataset presents its own challenges. Classical regression models require a priori selection of model form (linear, cubic, etc.) that is generally established through specific and extensive testing to check whether the data meets the assumptions and requirements of the statistical model. For our purposes, we required an analytical approach that (a) is capable of handling both numerical and categorical variables, (b) includes mechanisms for variable selection/importance rankings, (c) can handle non-linear relationships, and (d) produces interpretable results. Based on these criteria, we selected the random forest method for our analysis (Breiman 2001). Random forest builds an ensemble of classification and regression trees to make predictions. The algorithm constructs several hundred decision trees that each predicts the outcome based on a set of randomly confined observations and predictors. It then predicts the outcome by calculating the mean of the predictions of all individual decision trees. Several freely available software packages have been developed to aid the interpretation of random forests, and we used the forestFloor package as it has been previously deployed for similar purposes and provides good documentations (Welling et al. 2016).

For the analysis of the big picture data, we focused on the timing of nursery establishment as the very first activity that also strongly correlates with transplanting time and provides a good predictor of rice harvest, wheat planting, and wheat harvest (not shown). We further checked for variables that had a correlation factor of higher than 0.7 or lower than − 0.7 as they are known to decrease the accuracy of importance scores in random forest models. The only two highly correlated variables were cropped land and total landholding. We discarded the former but retained the latter, which thus represents both factors in the analysis.

In addition to the big picture survey variables, we estimated the monsoon onset for each survey point from Climate Hazards Group InfraRed Precipitation with Station Data (CHIRPS) 2.0 calculated based on agronomically relevant criteria: (a) the first day of two consecutive wet days cumulating in at least 35 mm rainfall and (b) no dry spell with a cumulative rainfall less than 5 mm within the 10 following days (Marteau et al. 2009). We also added pre-monsoon groundwater level from the Central Groundwater Resources Board from the year 2017 as a model predictor which was extrapolated through ordinary kriging to 0.05-degree resolution (Ministry of Water Resources 2016).

Next, for the factor importance ranking, we sought to identify the importance and relationship of each variable for shaping planting date (i.e., nursery establishment). For this purpose, we deployed the random forest model as implemented in the R package randomForest and plotted the variable importance score that is produced (Breiman 2001).

For the partial dependency plots, as to permit the inference of interactions between predictors and outcome, we used the forestFloor package (Welling et al. 2016). Partial dependency plots show the functional relationship between model input variables and model predictions with all other variables held constant. These plots show how the model predictions partially depend on the value of the input variable.

Representative trees are another helpful visualization. While the power of random forests comes from being able to generate numerous trees, they may not differ significantly from each other if sample sizes are large enough. Also, randomForest does not offer a simple way to visualize interactions between predictors. By choosing a tree that is statistically closest to all other trees, one can elucidate interactions among independent variables. This has, for instance, been suggested as a mechanism to deduce decision heuristics for medical practitioners (Banerjee et al. 2019). As such, we present a pruned version of a representative tree of our random forest model to provide a visualization of the main interactions among predictors as well as a sense of the factor hierarchy that governs planting dates.

#### Detailed dataset: analyzing discussion-based surveys with graph theory tools

The detailed dataset adds a qualitative dimension, and its results were used (a) to select variables that we subsequently included in the analysis of the big picture dataset, (b) to provide insights that bolster the interpretation of the results obtained from analysis of the big picture dataset, and (c) to identify missing or latent variables that were not directly captured in the big picture survey data, but which likely play a crucial role in explaining the reasons for untimely rice crop establishment. The detailed dataset is vital for developing conceptual models of systems because it is comprehensive, open-ended, and fully participatory. While results from the detailed dataset cannot be generalized to the regional scale because of its relatively small sample size, it does provide insights into the underlying factors associated with diverse establishment date outcomes. Building on this advantage, we relied on local agricultural experts to guide the village sampling strategy for stratification across precipitation, hydrology, and wealth gradients to capture a wide variety of possible cases. We used digital graph theory tools (igraph, GraphViz) to analyze scored causal diagrams created by the participants of each focus group discussion (Csardi and Nepusz 2006; Ellson et al. 2001).

These scored causal diagrams were digitized manually as a network graph through the GraphViz software package and exported for analysis with the igraph package in R (Csardi and Nepusz 2006; Ellson et al. 2001). For factor importance ranking, we calculated the weighted out-degree (sum of all outgoing arrows) for each factor of each individual farmer that scored the diagram. In the region, three different 2-week periods that are specified in the local calendar denote planting time: early (local calendar period called Rohini: 25th of May–7th of June, day of year 146–159), medium (local calendar period called Mirgishra: 8th of June–21st of June, day of year 160–173), and late (local calendar period called Adra: 22nd of June–5th of July, day of year 174–187). We recorded whether the farmers that scored the causal diagrams planted in each of the three periods. We then averaged the weighted out-degree scores of all farmers that planted rice nurseries in each 2-week period. Some farmers planted in multiple periods, mostly because they had plots on different land types and their scores were therefore counted in more than one period.

## Results and discussion

### Descriptive variable distributions of big picture dataset

**Fig. 3:**
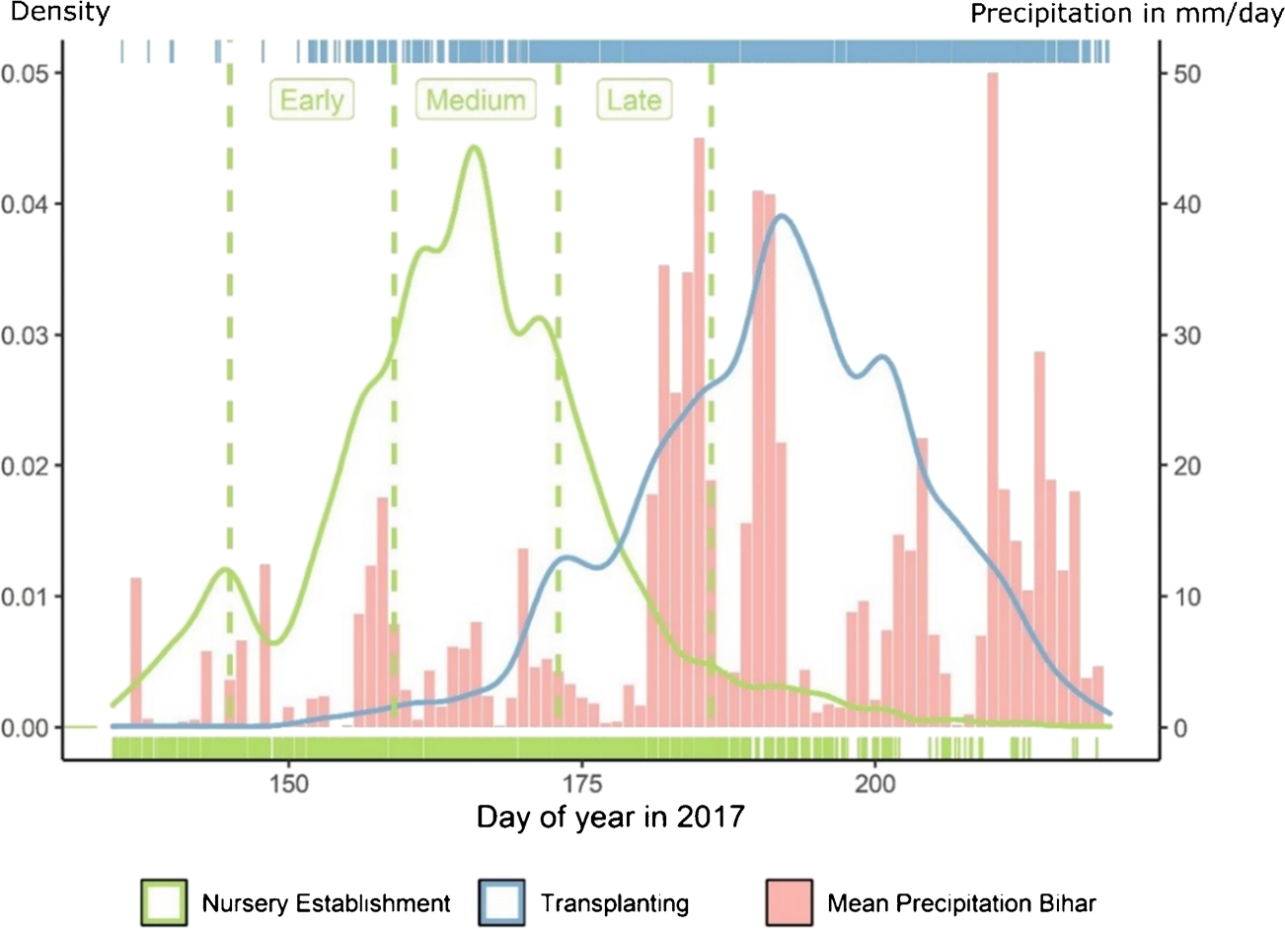
Distribution of rice nursery establishment and transplanting dates (*n* = 7597) in Bihar and Eastern Uttar Pradesh in 2017, including early, medium, and late time windows for nursery establishment and daily mean precipitation across the study area derived from CHRIPS. Farmers plant rice crops across more than 1 month. Peaks in nursery establishment occur with the onset of the early and late periods (denoting local farming calendars) as well as with the onset of sustained rainfall during the medium period. Transplanting activities follow nursery establishment patterns

For 2017, a normal monsoon year with onset around day of year 181, our data suggests that early planters (early period or before) are a distinctive minority (27%), while those falling into the medium category are the vast majority (50%), with late planters (late period or thereafter) in the minority (23%) (see Fig. 3). Our data shows three distinctive peaks in both the nursery establishment and transplanting distributions. For nursery establishment, the medium peak is likely related to the arrival of monsoon showers in the medium period. The early and late peaks likely relate to traditional farming calendars as they coincide with their starting day (see Section 2.2). Transplanting likely mirrors the pattern of nursery establishment through seedling age and monsoon arrival as a trigger for transplanting.

Some key numeric variables in our dataset have rather large outliers but are generally evenly distributed (not shown). Considering categorical variables, almost all farmers have access to irrigation, mostly from diesel-powered shallow groundwater wells, and grow wheat after their rice crop. The production in 2017 was widely perceived to be in line with the 5-year average. Surveyed farmers in the big picture survey reported to use the following heuristic for determining the timing for nursery establishment: calendar dates, pre-monsoon showers, and irrigation water availability, and, to a lesser extent, neighbors’ practices, seed availability, and weather forecasts. For transplanting, dominant self-reported heuristics were seedling age, calendar dates, irrigation water availability, rain arrival, and, to a lesser extent, labor availability. The influences on planting time distribution of the different self-reported heuristics can be seen in the different shapes of the distribution curves for nursery planting and transplanting (Fig. 3).

### Explaining planting date variability from a social-ecological perspective

**Fig. 4:**
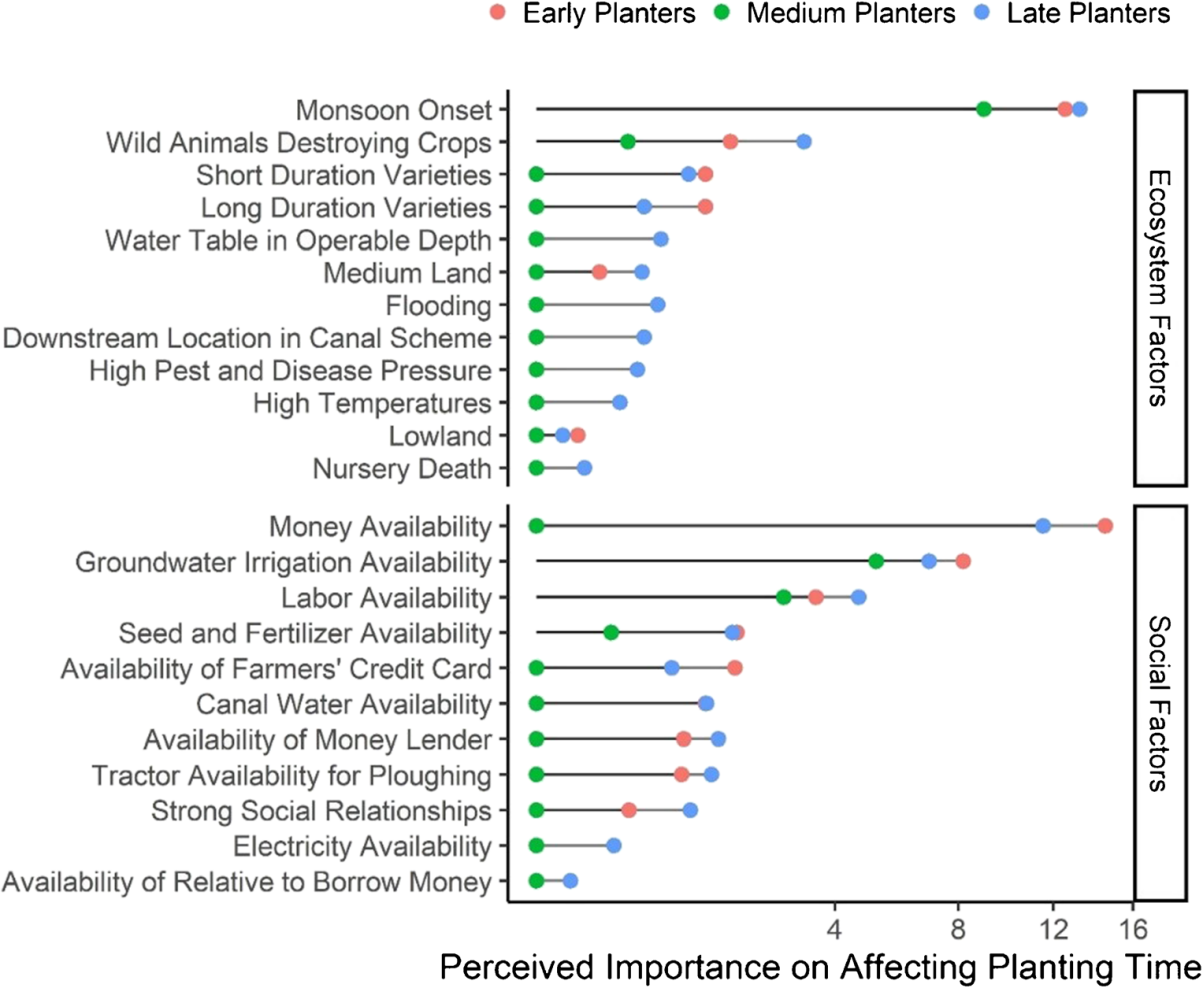
Factor importance rankings for the time of planting of the detailed survey. Water-related factors (both ecosystem and social) predominate. Wild animals, labor availability, and financial resources as well as timely availability of inputs further affect planting time

**Fig. 5:**
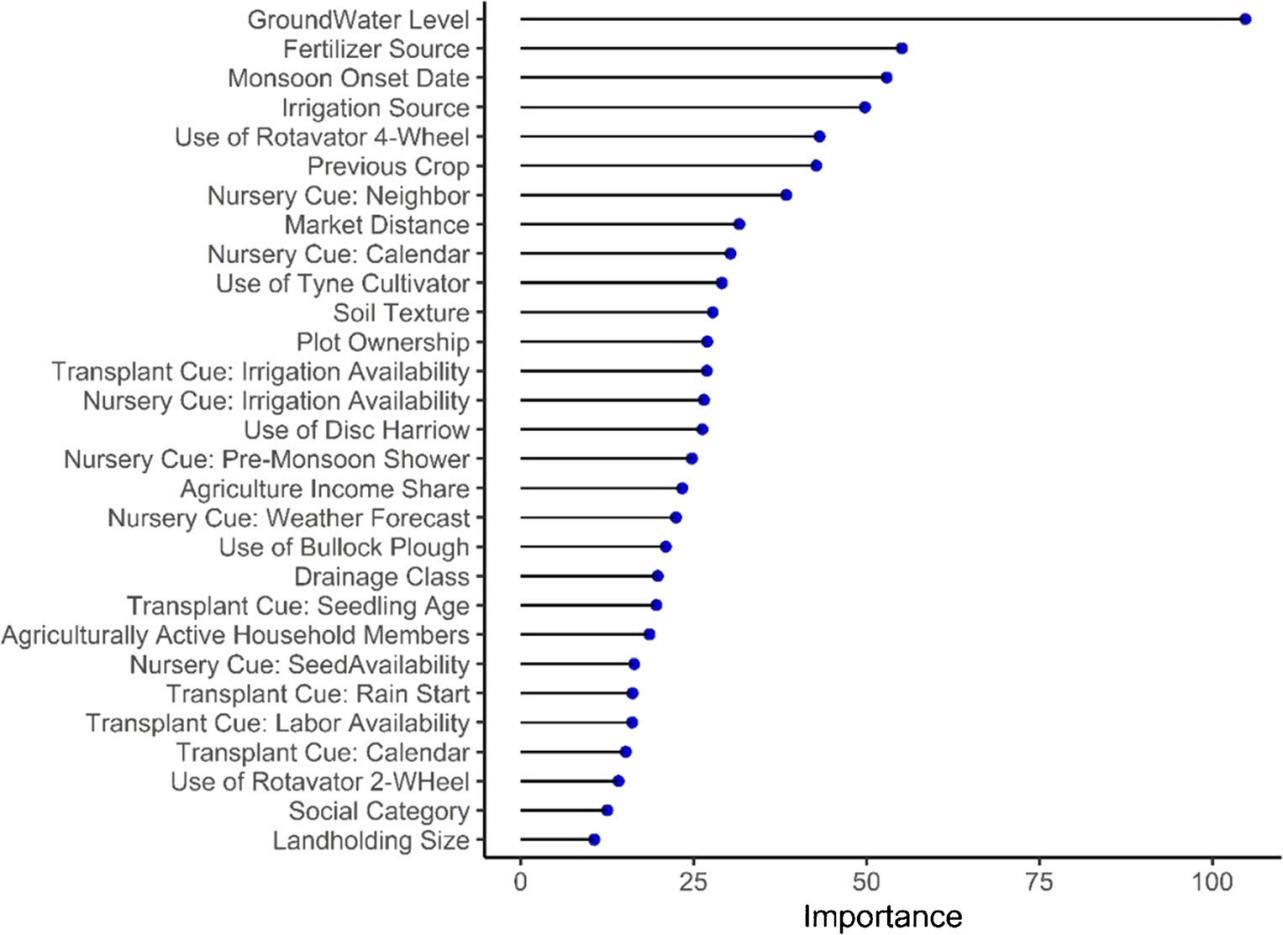
Factor importance rankings for the time of planting of variables in the big picture survey. Confirming the detailed survey, groundwater depth, monsoon onset, and irrigation source (all water-related factors) predominate. Other factors such as input types and availabilities, soil types, and decision-making factors further shape planting time

Monsoon onset date, results from both datasets suggest, plays a primary role in shaping the timing of rice planting (Figs. 4 and 5)—ostensibly to avoid costly groundwater irrigation as most farmers rely on costly and inefficient diesel-powered groundwater irrigation (Shah et al. 2018). This means that farmers who are socially constrained to access reliable and affordable irrigation face a trade-off between increasing their system resilience through early planting and increasing risks posed by potentially high irrigation expenses in the case of late monsoon onsets. Social factors, in general, act as secondary drivers of planting outcomes at the village and household levels, as farmers differ in access levels to agricultural inputs and services and investment capacity. For example, some villages reported that only one tractor is shared for plowing at transplanting so it requires ca. 30 days for the entire village to transplant. Likewise, labor for transplanting is often scarce as groups of migratory agricultural laborers are only available in each location at a given time so farmers need to pay a premium, wait, or actively import labor for transplanting. Similarly, farmers in remote villages require costly transportation to the nearest market to purchase quality seed and fertilizer. But, they may not have the capacity to mobilize the community and organize collective transportations for accessing inputs from market towns and thus rely on village-level shops that offer goods with varying quantities, qualities, and prices. In this way, secondary factors may cause rice planting delays even when water-related factors do not limit early planting.

Non-water-related ecosystem factors such as wild grazing animals (e.g. blue bulls [*Boselaphus tragocamelus*]; locally called *nilgai*) and pest and disease pressures play another important role. Pests, wild animals, and disease pressure did not emerge as a critical factor for contemporary decision-making in the big picture dataset. But, these factors were mentioned in the detailed survey in almost all villages as a barrier to early planting, especially if neighboring farmers were not planting in synchrony. Village-level commonalities in management also hint at the existence of “village tales” that shape a common understanding of optimal management that influences planting date decisions beyond variables that are collected in the big picture survey.

Our datasets demonstrate, for the first time, that water-related ecosystem factors play a primary role, but access to inputs and socio-economic stratification act as critical secondary factors, especially for resource-poor households. The big picture dataset also shows the limitation of small, detailed surveys that do not fully capture ecological regional variations such as variations in groundwater levels. To summarize the findings, Table 1 provides an overview of the characteristics that increase the probability of a farmer to plant early, medium, or late.

**Table 1:** Overview of the characteristics our data indicates to be associated with farmers that plant rice early, medium, or late in the Eastern Gangetic Plains

	Early planters	Medium planters	Late planters
Monsoon onset	Early monsoon onset	Normal monsoon onset	Medium or late monsoon onset
Blue bulls (*Boselaphus tragocamelus*)	Little to no blue bull presence	Little to medium blue bull presence	Blue bulls commonly present
Soil types	Heavy soils	Heavy to medium soils	Medium to light soils
Irrigation availability	Access to pre-monsoonal irrigation	Access to irrigation with monsoon onset	Only late access to irrigation (e.g., rental, canal dependent)
Tillage machinery availability	Timely availability of tillage machinery	Generally timely availability of tillage machinery	Little mechanization in village, long waiting times
Seed and fertilizer availability	Timely availability of seed and fertilizer	Generally timely availability of seed and fertilizer	Far away from markets and/or inadequate seed and fertilizer availability
Labor availability	Insufficient household labor and large landholding	Sufficient available household labor or small landholding	No timely labor available or renting out household labor
Collective action	High rate of other early planters	Some other early planters	Few to no other early planters

#### Landscape level: ecosystem factors

The water-related factors monsoon onset and irrigation availability appeared as critical factors in both the detailed dataset and the big picture dataset. In the detailed dataset, monsoon onset was rated as the most important factor in determining the date of rice planting for early, medium, and late planters. Monsoon onset was evaluated as the third most important variable in the random forest model which likely underestimates its importance as the big picture survey, on which the random forest was built, and only contains data from the year 2017. Monsoon onset is likely more important when explaining inter-annual variability, given that intra-annual variability in monsoon onset was limited in 2017 (data not shown).

**Fig. 6:**
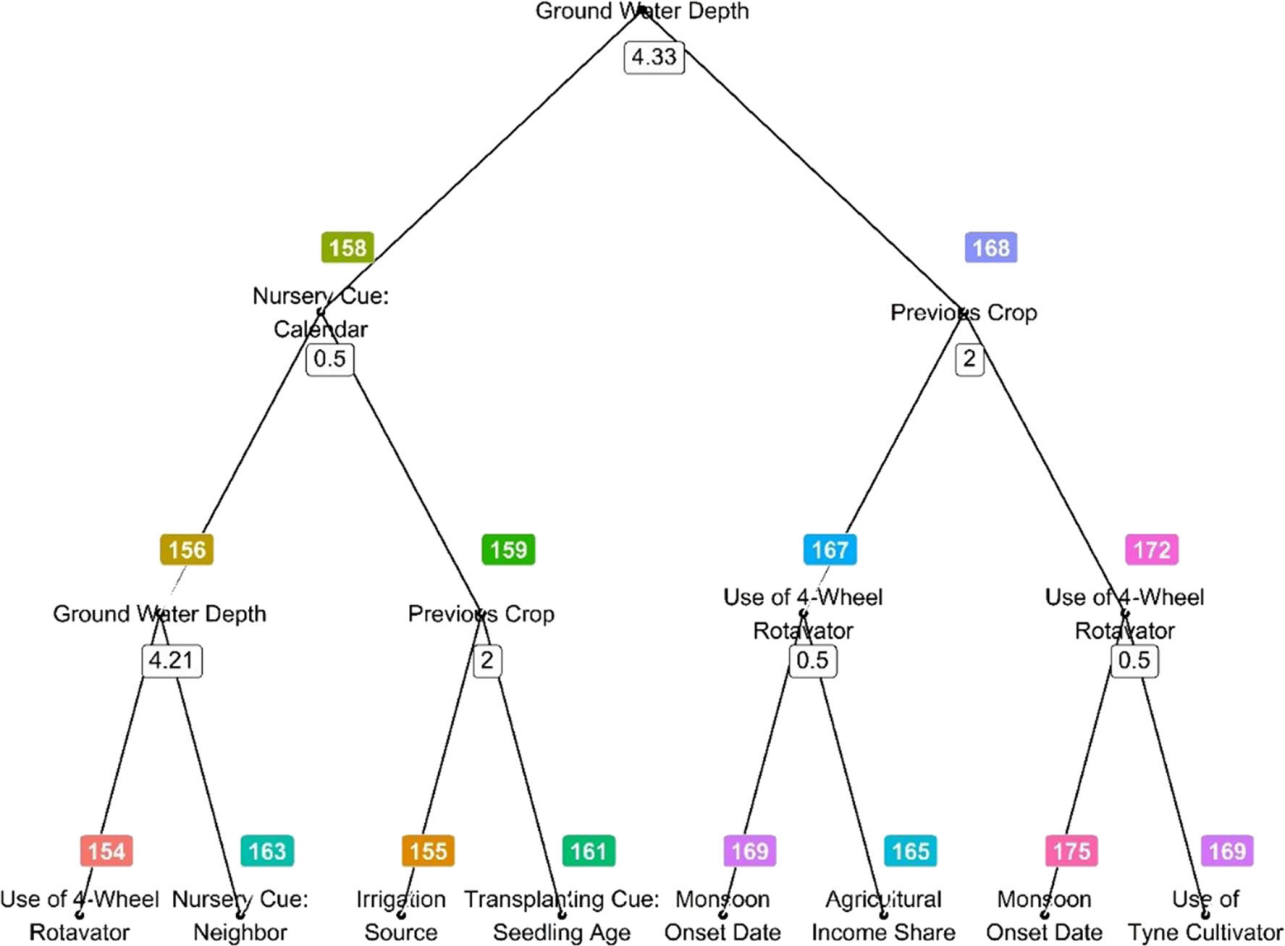
Representative tree (pruned) of the random forest model. This tree is closest in Euclidean space to most other trees and helps to derive heuristics from the model. Groundwater depth is a key separator for predicting planting time

**Fig. 7:**
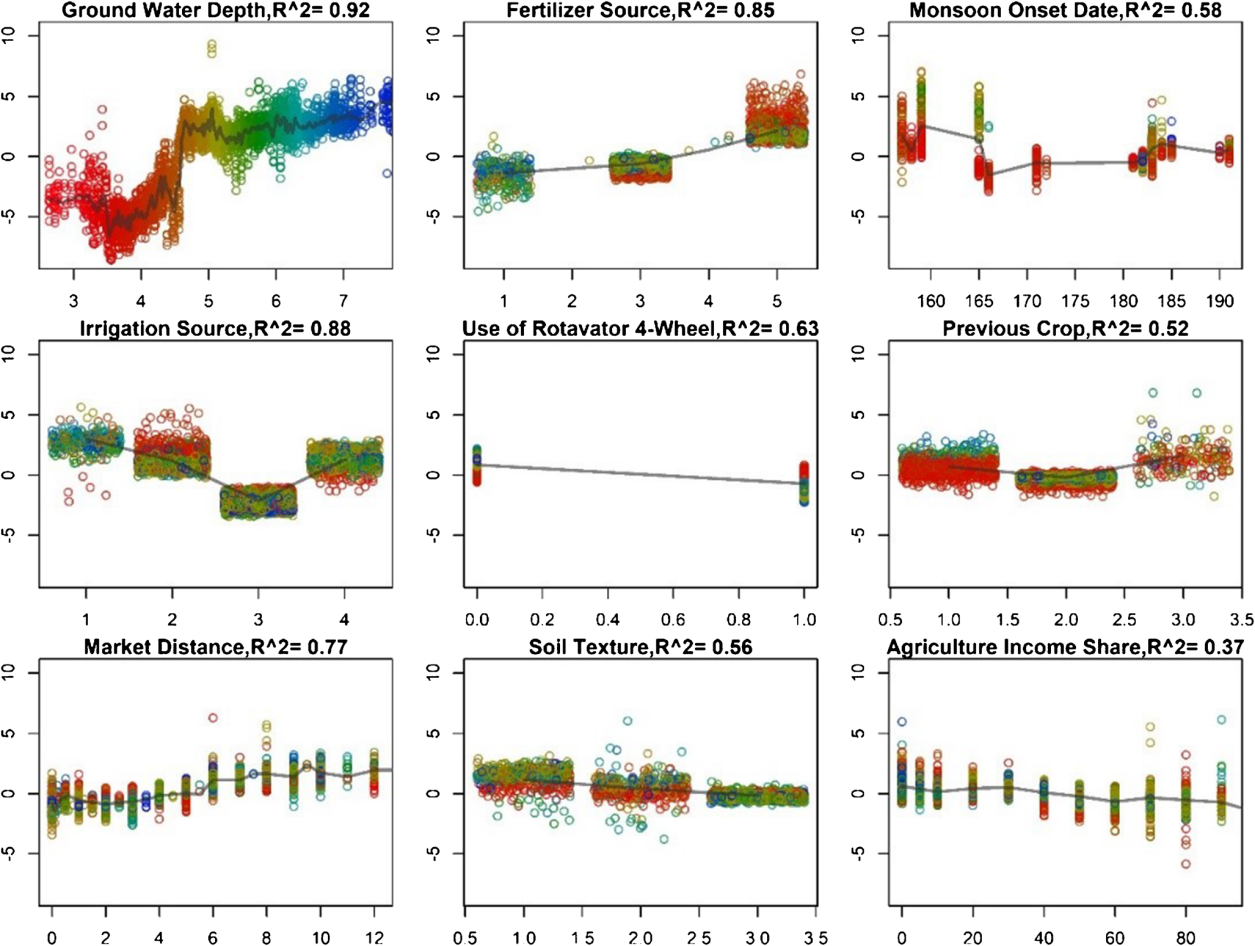
Partial dependency plots of the selected variables from the random forest model with observation points colored by groundwater level. Larger values on the *y*-axis indicate later planting and, for the groundwater level, clearly show a critical threshold at ca. 4.5 m

Groundwater depth scored second highest among the detailed dataset factor rankings and was the most important factor in the random forest model. The random forest results suggest that, at the regional scale, irrigation availability is primarily a function of shallow pre-monsoon groundwater levels, rather than machinery availability. The representative tree uses the groundwater depth variable as the first split and then further narrows down the predictions by splitting for different social variables as well as groundwater depth and monsoon onset at subsequent splits (Fig. 6). This splitting pattern further indicates that water-related variables are of primary importance, and other variables provide context on the degree to which early planting is (socially) constrained. The partial dependency plots further show that farmers tend to plant later if pre-monsoon groundwater levels pass a threshold of ca. 4.5 m (Fig. 7). This is likely explained by technical characteristics of centrifugal pumps that predominate the landscape. Centrifugal pumps, in theory, cannot lift water from more than 9.81 m below them, and in practice, friction losses commonly decrease this threshold even more (Kahnert 1993). In addition, the operation cost of centrifugal pumps increases sharply with deeper groundwater levels, which further amplifies the already high irrigation cost of diesel pumpset irrigation. At the village level, irrigation water in the region is generally made accessible by pump owners to other farmers on a pay-per-hour basis, so that variability in prices and availability can further constrain timely planting even where groundwater levels are in operable levels (Shah et al. 2018).

Access to canal infrastructure also emerged as an important factor. In canal areas, rice planting is often delayed. Farmers reported that they do not align rice planting with the monsoon onset, but with the opening of the canal system, which takes place after monsoon onset as monsoon rains are required to fill the canals. Farmers reported that farming in canal areas comes with the drawback that, in most regions, more water is released into canals than they can carry. Widespread flooding is the result, and farmers reported that fields are often submerged for several weeks at the start of the monsoon period. Farmers accordingly tend to raise submergence-tolerant seedlings and transplant them before flooding. The factors of flooding in the area are complex and, for instance, discussed in the study of Muthuwatta et al. (2017). Importantly, and mentioned by farmers during the focus group discussions, lack of transboundary cooperation between different states and countries plays a key role in causing the uncontrolled flooding.

The study revealed two further, non-water-related, ecosystem factors that feature a collective action component and appear to constrain timely planting in some places: wild animal grazing and pest and disease pressures. Wild animal grazing specifically refers to blue bulls, the largest Asian antelope which is widespread in the region. Farmers reported that they frequently destroyed crops as they graze in large herds. While the government allowed farmers to cull blue bulls in 2015, their status as a holy animal adds a layer of complexity to the issue. To defend against them, farmers install whistling tapes, scarecrows, and fences, but these methods are not always effective, and herds of blue bulls can still trample or graze on nurseries and young rice plants. While not directly affecting planting dates, the problems caused by blue bulls do increase the level of risk faced by rice farmers. Those that plant earlier than others are more at risk of damage as their nurseries and fields are more prominent and attractive for grazing.

The same logic applies to pest and disease pressures as has been observed in other locations (Tscharntke et al. 2012). Farmers reported that they cannot plant much earlier than neighboring farmers as their crops would otherwise succumb to heightened pest and disease pressures with pressure concentrating on the plots of early planters rather than being diluted across the landscape. Thus, early planting in a village is not only determined by access to resources, inputs, and technologies managed by single farmers. Rather, timely planting also depends on the ability and desire for synchronous early planting of neighboring farmers—a challenge further complicated by high and increasing levels of land fragmentation in the region (Keil et al. 2019).

#### Village and household level: social factors

At the village level, timely availability of cash, labor, machinery for land preparation and irrigation, seed, and fertilizer at adequate qualities and prices constitute additional factors that influence planting dates. In contrast to water availability, these social factors tend to have only a few high scorings per village and different factors appear to be more relevant depending on the village in question, resulting in lower scores of the factor importance ranking across locations from the detailed dataset (Fig. 4). Social factors were also attributed less importance by the random forest model, which may be because there were fewer social factor variables in the big picture survey and post-sampling social stratification was not feasible due to the large sample size and random household selection in each village. The factor rankings suggest that, at the regional scale, social variation at the village level (e.g., market distance, machinery availability) is more important than household-level differences within a village. Nevertheless, farmers did report that households with a low socio-economic standing generally face more hardship for accessing inputs. Altogether, it seems that not only availability of a given input is important; rather, farmers’ capacity to purchase and use the input effectively is also crucial. This metric, however, was not captured in the big picture survey and therefore also not reflected in the random forest model.

Similarly, farmers reported that labor constraints are increasing and especially impactful for households with larger landholding who tend to rely on hired labor that is difficult to find after the onset of the monsoon when most labor is then engaged in transplanting rice on their own plots. Larger landholders with cash available thus tend to plant rice earlier than their neighbors to avoid labor shortages. Farmers in remote villages also noted that, in addition to machinery inputs and labor, quality seed and fertilizer at subsidized rates are not always available on time. For timely supplies, remote farmers would have to travel to distant market towns which is costly. And, the lack of collective action models complicates the mobilization of collective transportation, which would reduce individual costs.

In addition, finance to purchase inputs and labor scores highest among the social factors as a constraint to timely planting in the detailed survey. This is likely to reflect the difficulty to avail cash when needed. Some farmers reported that they use a credit card that is provided and subsidized by government initiatives, but most farmers borrowed cash from relatives or local moneylenders. Although credit sources were not explicitly recorded in the big picture survey, metrics for market integration such as distance to market and fertilizer sources may be good proxies. However, moneylenders and microfinance alone cannot provide a solution to these constraints. We therefore focus our subsequent discussions on increasing profitability, resource use efficiency, and business models for input and service provisioning that cater to different farmers’ needs.

Soil and crop variety types also emerged as variables affecting timely planting in the detailed survey. Farmers indicated that different drainage classes (land types) tend to prompt different planting dates and variety choices. Differences in soil moisture retention lead to a shorter growing season on land with faster drainage and thus shorter-duration rice varieties and later planting. If irrigation was available, however, farmers reported that, on preference, they made use of the early drainage of upland areas to plant vegetables after short-duration rice varieties. Many lower lying lands, on the other hand, were also prone to waterlogging and thus required earlier planting of longer-duration varieties so that transplanting and harvesting could both take place at desired soil moisture levels.

### Enhancing farmers’ capacity for timely rice planting in the Eastern Gangetic Plains

#### Ecosystem factors

Our results suggest that water-related factors most strongly shape rice planting date patterns in the Eastern Gangetic Plains. Research suggests that improving unreliable and expensive irrigation infrastructure is a pre-requisite for sustainable agricultural development in the Eastern Gangetic Plains (Shah et al. 2018). Two major initiatives are currently being promoted by governments and development organizations in the Eastern Gangetic Plains: (i) a centrally led initiative to electrify the countryside and (ii) a subsidized effort to scale-out solar irrigation. Both are promising initiatives that may significantly reduce the operational costs of pumping. The risk of depleting groundwater in the Eastern Gangetic Plains—that generally accompanies groundwater development—has been largely dispelled by previous studies (Muthuwatta et al. 2017; Shah et al. 2018). If successful, many more farmers could gain pre-monsoon irrigation access because of these initiatives. They could be empowered to plant their crops in a timelier manner and reduce the level of climatic risks associated with delayed monsoon onset.

Spatial targeting in conjunction with supplementary investments in existing technologies could potentially enhance the effect of the regional initiatives to scale out low-carbon and affordable irrigation technologies. Our results suggest that areas with pre-monsoon groundwater tables below 4 m require alternatives to centrifugal pumps, such as electrically or solar-powered submersible pumps to provide affordable pre-monsoonal irrigation (Shah et al. 2018). But, in areas with pre-monsoonal water tables at depths less than 4 m, increasing access to efficient diesel pumpsets may prove a more cost-effective approach in the near term (Urfels et al. 2020). For solar, the current subsidy schemes substantially reduce the capital investment costs for solar-powered irrigation systems. Its manufacturing costs are also decreasing and may enhance economic viability for future deployment, potentially superseding diesel pumpsets in the Eastern Gangetic Plains over the next decades (Shah et al. 2018). Although several studies have been conducted on sustainable groundwater use in the area, including managed aquifer recharge systems, specific recharge processes and detailed aquifer maps remain a knowledge gap that needs to be filled to confidently design sustainable groundwater use scenarios (Reddy et al. 2020).

Wild grazing animals and pest and disease pressures further shape farmers’ capacity for timely planting as individual early planted plots are more likely to face concentrated biotic pressures. These dynamics, although crucial for agricultural practices and critical for ungulate conservation, are not easily quantified (Tscharntke et al. 2012; Prestele and Verburg 2019). Development of new survey instruments and use of new analytical methods are likely required to capture them. These shortfalls highlight remaining challenges in ecosystem services research and the design of agricultural development pathways that align with broader conservation targets. In addition, our findings highlight that building agroecosystem resilience involves developing solutions to collective action problems which need to be addressed at larger spatial scales than the plot or household level (Prestele and Verburg 2019).

#### Social factors

A strong agricultural goods and service economy (i.e., market integration) appears to contribute to farmers’ ability to plant early. Specifically, our data corroborate evidence that access to labor, tillage, and subsidized seed and fertilizer markets frequently prevents timely planting and requires innovative solutions (Keil et al. 2019; Shah et al. 2018). Strategies to strengthen land preparation machinery service markets could consider the spatial gradients of commercial orientation and mechanization both at the community level and at the household level. Subsequently, it is not necessarily large-scale infrastructure projects, but locally targeted investments that are tailored to farmers’ needs, that may allow farmers to better tackle specific bottlenecks and increase their flexibility in making decisions.

Innovating agricultural input markets to support timely planting will inevitably reveal synergies and trade-offs with other sustainability targets. These should be considered by policy makers to increase investment effectiveness, as has been recently shown in other places. For example, zero-tillage wheat, which is mainly provided on a pay-per-hour/ha service by private machinery owners, has been shown to provide “win-win” scenarios for farmers, but adoption is largely inhibited by a lack of awareness among poorer farmers. The possibility to use tractors for both zero-tillage wheat and direct seeded rice planting may further enhance attractiveness to farmers and service providers. But while mechanical rice planting continues to face problems, improvements in custom hiring of tillage services may provide welcome synergies for timely planting until more effective models of mechanized rice planting that are also attractive to poorer farmers are positioned for large-scale adoption. In the meantime, tillage service providers may play a crucial role in coordinating synchronous planting, hence saving fuel by reducing travel between villages and assisting farmers to overcome landscape-level ecological pressures. Highlighting the labor-saving benefits of mechanical planting is another consideration. But, just as with irrigation, investing in human capital and strengthening the supporting industry of mechanics, vendors, and other private sector actors along the value chain are required to leverage cross-sectoral benefits and provide potential employment options for agricultural laborers that, in some areas, may be put out of business.

Our results further highlight the value of developing spatial datasets of social variables to build resilient agroecosystems. Spatial variation of agroecosystems matters for sustainable agricultural development pathways because nuanced differences in the social-ecological setup produce different decision-making patterns. For example, investments in irrigation should be guided by differentiations between upstream/downstream and flooded/non-flooded canal areas, submersible pumps, and centrifugal pumps with pre-monsoon groundwater levels below or above 4.5 m. Furthermore, data on access to inputs and the quality and timeliness of service provision can be collected at the household or village level. The number of tractors per capita at the village level might, for instance, be a useful overall measure, but ideally, data on all key inputs should be collected.

Embellishing climate services and dynamic planting date advisories constitutes another channel through which farmers’ capacity for timely planting can be enhanced. Both farmers and agronomists use cropping calendars to characterize planting and harvesting times of crops in different agroecological zones. Streamlining activities such as application of irrigation, fertilizers, pesticides, and herbicides into these calendar-oriented formats that align with the phenological development of crops can consolidate research findings for effective communication with end-users (Subash and Gangwar 2014). Our study showed that farmers in the region use a local calendar of 2-week periods for temporal orientation. We therefore recommend that farmers’ temporal heuristics could be integrated and consolidated in extension efforts to ease communication barriers and enhance the potential for farmers’ adoption of resilience enhancing practices.

### Building resilient agroecosystems through timely planting

Building sustainable and resilient agroecosystems constitutes a major global policy target, but understanding regionally specific incentive structures, factors, and barriers to catalyze transformative changes remains a crucial challenge. Our findings provide new methodological and practical insights on agroecosystems as social-ecological systems with special regard for climate change and food security. Integrated research frameworks in the field of food security and climate change have been continuously refined for more than two decades. Digitalization and ongoing advances on spatial and detailed data collection, analytics, and decision support systems can and must be leveraged for fine-tuning the evidence base by crystallizing regionally specific elements that influence behavior and outcomes. Critical future steps include (a) producing and comparing results to other regions and social-ecological systems to better understand their differences, (b) including the factors and processes that our analysis unveiled in regional modeling and integrated *ex-ante* assessments, (c) leveraging synergies and trade-offs with other sustainable agricultural technologies, (d) increasing the availability of high-resolution spatial data, and (e) developing targeted and evidence-based programs with a spatially specific and holistic theory of change (Van Noordwijk 2019).

In addition to the research needs outlined above, policy makers in the Eastern Gangetic Plains can support farmers through multifaceted efforts to improve access to irrigation, labor, tractors, and seed and fertilizer, as well as conserving wild animals in ways that reduce risk of crop damage. Specifically, metered electricity connections and submersible pumps should primarily be encouraged in areas with pre-monsoonal groundwater tables below 4 m. Solar irrigation systems should be targeted for centrifugal pumps in areas with shallower groundwater tables. Similarly, scale-appropriate agricultural mechanization with pro-poor models of service provision to facilitate smallholder access to machinery requires acceleration in areas of low technology penetration (Paudel et al. 2019). Furthermore, seed and fertilizer markets as well as other inputs should be made more easily available to remote villages, e.g., through stimulating private sector extensions of agricultural input providers. And, conversely, policy should also focus on encouraging improved resource use efficiencies to reduce input needs. Collective action models for achieving more synchronous planting as well as early warning systems should be established to avoid damages from wild animals, pests, and diseases with information disseminated through existing extension networks and the private sector.

### Studying agroecosystem at the regional scale through a mixed-methods social-ecological approach: challenges and opportunities

Our mixed-methods approach identified key issues and dynamics for timely rice planting in the Eastern Gangetic Plains. Using machine learning tools to analyze a combination of big picture and detailed survey datasets assisted in (i) uncovering diverse pathways through which different variables shape farmers’ planting behavior, (ii) highlighting non-linear critical thresholds of important variables (e.g., groundwater level) and interaction effects between variables (e.g., where certain social conditions reduced or amplified the effect of groundwater level), and (iii) identifying factors that the big picture surveys do not reveal, such as grazing wild animals and unwillingness to plant early due to heightened pest and disease pressure—both of which contain properties of classical collective action problems. Our approach can be used for future studies to build systematic evidence for key sustainability challenges in complex social-ecological systems amidst increasingly rapid global environmental change. The approach offers a balance of analytical depth and size of the inference space. Ideally, big picture surveys should follow and be informed by the detailed surveys, which should be taken into consideration for future study designs. We found that potentially valuable variables, especially social variables, that measure the timeliness of village-level service provision were not included in the big picture survey. We can only recommend for these to be included in future data collection efforts and act as a tool to stratify the sampling. We aimed to show that such an approach can effectively complement the shortcomings of the two types of datasets and methods of inference each one makes possible, and thus contribute to spanning the gap between regional programming and context-specific interventions. These will be increasingly important to ensure a sustainable transition of the food system in the future.

Major challenges also pertain to the conceptual development of agroecosystems as social-ecological systems and resilience in agricultural development. While several authors have developed social-ecological systems frameworks for sustainable agricultural development and pointed at required modifications, few examples of their operationalization exist (Lescourret et al. 2015). From a user-centered perspective, farmers’ capacity to adapt to the environmental change and build resilience against environmental shocks such as late monsoon onsets depends on their levels of access to resources (e.g., water) and markets (e.g., labor, fertilizer) and is partially mediated through technology (e.g., pumps, tractors). The system boundaries can be scaled from the farm to the community and to the landscape level—with important interactions between the scales and different possibilities for interventions at each scale. From this perspective, timely planting requires flexibility regarding the use of water and other agricultural inputs. At the landscape level, this means that farmers’ resilience hinges on the response of the resource base (e.g., water resources) and input markets in the face of external shocks (e.g., multiseason drought effect on groundwater or oil market price fluctuation for tractor service provisions). Enhancing farmers’ timely access to inputs, while coupling these efforts with management practices to improve resource use efficiency, enables them to raise profits and yields amidst progressive global change, while enabling access to water resources beyond critical technological thresholds buffers farmers against moderate environmental shocks. But, our research cannot immediately be extended to drastic disturbances in the climate system as the effects on input markets and the resource base might differ and invoke other tipping points. Achieving agroecosystem resilience beyond the level discussed in this study requires further research to reveal the effects of more complex and more drastic global change processes and find entry points to increase farmers’ resilience against such disturbances (Schipanski et al. 2016).

## Conclusion

Smallholders in the Eastern Gangetic Plains rely on timely rice planting for building resilient agroecosystems amidst progressive environmental change. In this study, we used a novel, social-ecological systems informed, mixed-methods approach. This approach revealed for the first time that farmers’ capacity for timely planting is primarily predicated on the timely availability of pre-monsoonal irrigation, while social factors such as timely access to farm inputs and machinery act as secondary constraints for timely planting for many farmers in the region. In addition, absence of collective action in the form of synchronous rice planting to reduce pressure from pests, diseases, and grazing animals on individual plots emerged as an additional, but not quantified constraint.

Based on these finding, we argue for advanced research to support spatial targeting of pro-poor investments to overcome spatially explicit barriers to timely use of irrigation, machinery, and farm inputs. Doing so will require new data collection efforts that quantify the spatial structure of these barriers. In addition, finding models that can solve collective action problems, at times perhaps through creation of new service models in the private sector, and improving resource use efficiencies to make farmers less reliant on external resources will also enhance farmers’ capacity for timely planting and thus agroecosystem resilience. Lastly, the sustainability of some of these interventions also depends on the resilience and sustainability of the resource base as well as input, machinery, and labor markets. Understanding these will require research beyond the agroecosystem, e.g., on the food system, on how these system components behave amidst global environmental change.

## Data Availability

The datasets generated and/or analyzed during the current study are available from the corresponding author upon reasonable request.
